# Impact of Drug-Resistance Polymerase Mutations on the Replication of HBeAg-Positive and HBeAg-Negative Hepatitis B Virus Strains in Vitro

**DOI:** 10.5812/hepatmon.6131

**Published:** 2012-06-30

**Authors:** Frank Tacke, Elham Shirvani-Dastgerdi

**Affiliations:** 1 Department of Medicine III, University Hospital Aachen, Aachen, Germany

**Keywords:** Hepatitis B e Antigens, HBV, Precore, Basal Core Promoter, Drug Resistance

Approximately 350 million people are chronically infected by the hepatitis B virus (HBV) worldwide, and an increasing percentage of these patients present themselves with HBeAg-negative disease. In many parts of the world, such as Southern and Central Europe or also Iran, HBeAg-negative hepatitis B nowadays accounts for the majority of cases [1][2]. HBeAg-negative hepatitis B develops spontaneously during chronic infection through mutations in the precore or core promoter region of the HBV genome, either ablating HBeAg expression by a stop codon in the precore region (called ‘precore’ [PC] mutation) or suppressing transcription of the precore RNA by a basal core promoter variation (termed ‘basal core promoter’ [BCP] mutation) [3]. Patients with HBeAg-negative chronic hepatitis B have commonly more advanced liver disease than HBeAg-positive patients, and the likelihood of spontaneous remission is very low. The distinct presence of PC or BCP mutations became a focus of research interest due to their important clinical consequences. It has been demonstrated that PC / BCP mutations are associated with a significantly increased risk to progress to liver cirrhosis and hepatocellular carcinoma (HCC) during the natural course of HBV infection [4][5]. Moreover, these mutations are independent risk factors for the development of acute-on-chronic liver failure [6] and patients with an acute-on-chronic liver failure with PC mutant virus have an increased risk of death [7]. The BCP mutation is specifically associated with a significantly increased risk of HCC (3.79 fold increased risk of HCC compared with HBV without mutations) and it is detected approximately 10 years before the diagnosis of HCC suggesting that BCP mutation is an early event in hepatocarcinogenesis [8].

**Figure 1:**
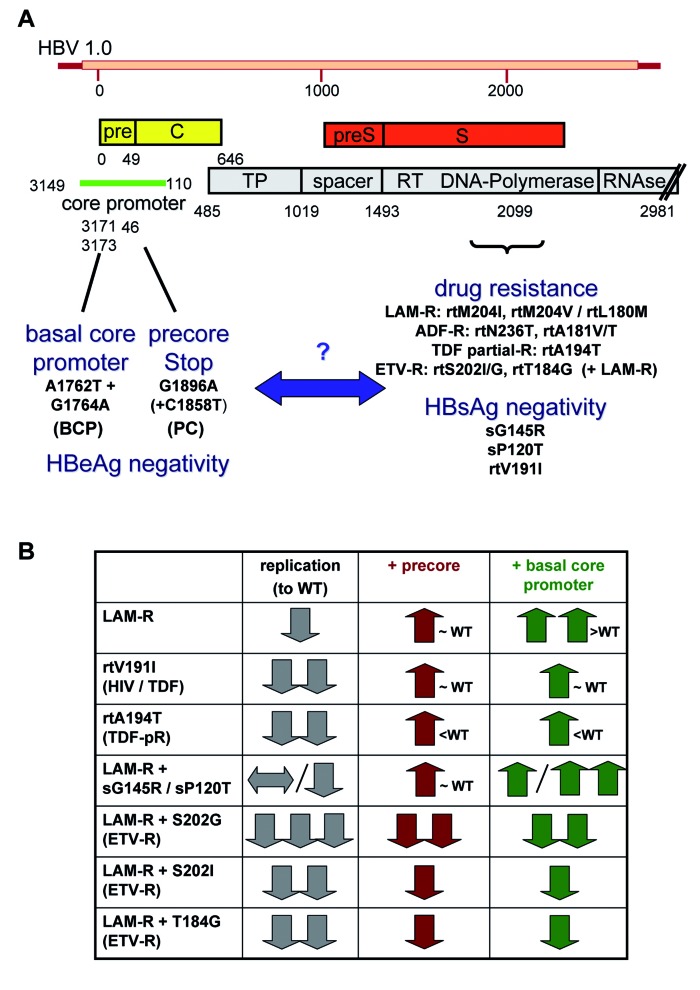
Impact of Polymerase/Surface-Antigen Mutations on the Replication of HBeAg- negative Hepatitis B Virus (HBV) Strains in vitro. (A) Schematic depiction of the 1.0-fold HBV genome, showing mutations suppressing HBeAg expression in the basal core promoter (BCP) or precore (PC) region as well as mutations in the polymerase or overlapping surface genes, which are related to drug resistance and/or HBsAg negativity. In vitro replication systems have been developed to study functional consequences of HBeAg negativity (BCP/PC mutants) on viral replication of polymerase/surface-antigen mutants. (B) Schematic and simplified summary of several in vitro studies from our group, in which we analyzed the impact of either wildtype core, precore and basal core promoter mutations on the replication efficiency of various polymerase/surface mutations. References are given in the main text. Abbreviations: ADF, adefovir; eTV, entecavir; LAM, lamivudine; TDF, tenofovir; -R, resistance

For antiviral therapy, pegylated interferon and five nucleoside/nucleotide analogues (lamivudine, adefovir, entecavir, telbivudine and tenofovir) are approved at present [9]. Although pegylated interferon can be applied in a finite therapeutic course of 48 weeks and may induce HBsAg level decrease or even HBs seroconversion in some patients, it is rarely used in HBeAg-negative patients because of considerable side effects, several contraindications and the overall low response rate, especially in genotype D infected patients [10]. Treatment with nucleoside/nucleotide analogues is generally safe and well tolerated, but usually requires a long, oftentimes life-long therapy, as relapses after discontinuation of therapy regularly occur [9]. Especially the “first generation” antiviral drugs lamivudine and adefovir, which are still widely used worldwide, bear a high risk of selecting mutations in the HBV polymerase region that confer drug resistance. The most common mutations associated with lamivudine resistance occur in the YMDD motif of the C domain of HBV polymerase region causing a methionine change with either isoleucine (rtM204I) or valine (rtM204V). In most of the cases rtM204V mutation is not present alone but linked with a leucine to methionine exchange at position 180 (rtL180M) [3]. From a molecular level, it is well known that many drug-resistance associated polymerase mutations, such as lamivudine resistance, reduce the replication efficacy of HBV. Several in vitro systems have been developed which allow studying the replication level of wildtype and mutated HBV strains, for instance by using transient transfections of replication-competent HBV plasmids or baculoviruses [3]. Given the exceptional clinical importance of HBeAg negativity, we tested the hypothesis that PC or BCP mutations influence the replication capacity of polymerase mutants (Figure 1A). Remarkly, we and others were able to demonstrate that PC and BCP mutations can enhance the replication fitness of lamivudine-resistant HBV strains (Figure 1B) [11][12]. Thus, the concomitant presence of PC or BCP mutations might render HBeAg-negative patients at a particular risk when developing drug resistance. These principal findings provoked a series of additional studies, revealing that the effects of PC/BCP mutations vary remarkably between different polymerase mutants (Figure 1). In case of lamivudine resistance, PC mutations enhanced replication of polymerase mutants to WT level, and BCP mutations even further increased their fitness [11]. Similar findings were observed with the rtV191I polymerase mutant that is associated with lamivudine resistance and HBsAg-negative hepatitis B [13]. Furthermore, mutations in the envelope protein, i.e. the sG145R or sP120T substitutions, that affect the overlapping polymerase gene, increased by itself and in conjunction with PC or BCP mutations the replication efficacy of lamivudine-resistant HBV mutants [14]. On the other hand, PC or BCP mutations had very modest influence on the rtA194T polymerase mutation, which has been associated with reduced susceptibility towards tenofovir in vitro and in HIV-HBV-coinfected patients (Figure 1B) [15][16]. Entecavir is a relatively new and very well tolerated compound with high antiviral efficacy that is regarded as first-line therapy for HBeAg-negative patients [9][10] BH. In treatment-naïve patients, the rate of drug resistance is very low; however, patients pretreated with lamivudine or patients with existing lamivudine resistance are at high risk of developing entecavir-resistance [17]. On a molecular level, entecavir-resistance requires lamivudine-resistance (rtM204V/rtL180M, rtM204I) plus an additional polymerase mutation, mostly rtS202G, rtS202I or rtT184G substitutions [18]. In functional assays, all entecavir-resistant polymerase mutants showed a very low replication level in vitro [19]. The effects of PC/BCP mutations on the replicative capacity of entecavir-resistant strains appeared to be particular compared to other drug-resistant mutants. Addition of either PC or BCP mutations to rtS202G mutants (plus lamivudine resistance) only very moderately enhanced the reduced replication. More surprisingly, PC and BCP mutations did not significantly change the replicative capacity of rtS202I or rtT184G mutants (plus rtL180M/rtM204V lamivudine resistance) [19]. Collectively, these data demonstrated that HBeAg-suppressing PC or BCP mutations cannot restore the strongly reduced replicative capacity of entecavir-resistant HBV mutants to wildtype level, although they moderately increased replication of rtS202G combination mutants. This underlines that entecavir-resistance differs from lamivudine-resistance alone, corroborating that entecavir is a safe option for HBeAg-negative patients [9].

In conclusion, HBeAg-negative hepatitis B represents an increasing clinical challenge and often requires long-term therapy with nucleos/tide analogues, e.g. entecavir or tenofovir. Functional consequences of HBV mutations can be studied in vitro, using transient transfection of replication-competent HBV vectors. Importantly, while polymerase mutations associated with drug resistance usually decrease the replication efficacy of HBV, simultaneous presence of PC or BCP mutations may substantially enhance the replication of drug-resistant HBV mutants. These studies are anticipated to allow a better understanding of functional consequences of distinct mutational patterns and may help to guide antiviral strategies.
